# Early-stage non-alcoholic fatty liver disease in relation to atherosclerosis and inflammation

**DOI:** 10.1016/j.clinsp.2023.100301

**Published:** 2023-11-11

**Authors:** Si-hua Tan, Xiao-li Zhou

**Affiliations:** Department of Cardiology, The First Affiliated Hospital of Chongqing Medical University, China

**Keywords:** Atherosclerosis, Coronary heart disease, Non-alcoholic fatty liver disease, Liver fibrosis, Inflammation

## Abstract

**Background and aims:**

Non-alcoholic fatty liver disease (NAFLD) is a multisystem disease closely linked to cardiovascular disease (CVD). This study aims to investigate the connection between early-stage NAFLD and atherosclerosis, as well as the correlation between liver fibrosis and coronary heart disease while exploring underlying inflammatory mechanisms.

**Methods:**

In this retrospective study, the authors analyzed data from 607 patients who underwent both coronary computed tomography angiography (CCTA) and abdominal ultrasonography (US). Logistic regression was utilized to examine the association between NAFLD and atherosclerosis, while mediation analysis was conducted to explore whether inflammatory markers mediate the link between liver fibrosis and coronary artery disease.

**Results:**

Among the 607 patients included, 237 (39.0 %) were diagnosed with NAFLD through ultrasonography. After adjusting for traditional cardiovascular risk factors, ALT, and AST, NAFLD demonstrated a significant correlation with carotid intimal thickening (1.58, 95 % CI 1.04‒2.40; *p* = 0.034) and non-calcified plaque (1.56, 95 % CI 1.03‒2.37; *p* = 0.038). Additionally, fibrosis predictive markers, including FIB-4 > 1.3 (1.06, 95 % CI 2.30‒5.00; *p* = 0.035) and APRI (6.26, 95 % CI 1.03‒37.05; *p* = 0.046), independently correlated with coronary heart disease after adjusting for cardiovascular risk factors. Conversely, among systemic inflammatory markers, only the neutrophil-to-lymphocyte ratio (NLR) and systemic inflammatory response index (SIRI) are independently associated with coronary heart disease. ROC curve analysis indicated that combining predictive fibrosis markers or inflammatory markers with traditional cardiovascular risk factors enhanced the predictive accuracy for coronary heart disease. Mediation analysis revealed that NLR fully mediated the effect of liver fibrosis on coronary heart disease.

**Conclusion:**

NAFLD is associated with carotid intimal thickening and non-calcified plaque, suggesting an increased cardiovascular risk. Furthermore, liver fibrosis independently increases the risk of coronary heart disease in the early-stage NAFLD population, and inflammation may play a fully mediating role in the effect of liver fibrosis on coronary heart disease. Early intervention is crucial for NAFLD patients to mitigate future major adverse cardiovascular events.

## Highlight

• Non-alcoholic fatty liver disease was associated with carotid intimal thickening and non-calcified plaque, highlighting an elevated cardiovascular risk.
• NAFLD fibrosis is an independent risk factor for CHD.
• Inflammation potentially serves as a complete mediator in the connection between NAFLD fibrosis and CHD.

## Introduction

Non-alcoholic fatty liver disease (NAFLD) is the most common chronic liver disease worldwide, with a global prevalence of 24 %.^1^ It includes a spectrum of liver diseases ranging from simple fatty liver, non-alcoholic steatohepatitis (NASH), to cirrhosis and hepatocellular carcinoma. There is growing evidence that NAFLD is a multisystem disease that may increase the risk of developing type 2 diabetes, cardiovascular disease, and chronic kidney disease.^2^ In addition, liver fibrosis is the strongest predictor of future liver-related events in patients with NAFLD and can be assessed by liver biopsy, imaging techniques, or noninvasive biomarkers. Currently, liver biopsy and histology remain the gold standard but are difficult to perform widely in the population because they are invasive procedures. In contrast, fibrosis predictive markers, such as the NAFLD fibrosis score (NFS) and fibrosis-4 (FIB-4) index, have become the most commonly used screening indicators of fibrosis severity in clinical practice due to their simplicity and non-invasive nature.^3^

Cardiovascular disease (CVD) stands as the primary cause of mortality among NAFLD patients. Within CVD cases, Coronary heart disease (CHD) makes up around one-third to half of the total instances, marking it as the leading cause of death among adults.^4^ A meta-analysis study has unearthed a connection between NAFLD and an escalated long-term risk of fatal or non-fatal CVD events, independent of cardiometabolic risk factors. This holds particularly true in the context of advanced fibrosis stages.^5^ Additionally, a separate cohort study has highlighted the correlation between liver fibrosis and numerous cardiometabolic disease risk factors.^6^ Therefore, it is still unclear whether the severity of fibrosis in early-stage NAFLD, including simple fatty liver and NASH, is an independent risk factor for CHD, which is of great significance for early clinical identification and intervention of high-risk patients and may improve the prognosis of such patients. At present, the mechanism by which NAFLD increases CVD risk is not fully understood and may include insulin resistance, oxidative stress, systemic inflammation, dyslipidemia, etc.^7^ As the core of all stages of atherosclerosis, it remains unclear whether inflammation plays a bridging role between early-stage NAFLD fibrosis and CHD.

This study explores the association between early-stage NAFLD and atherosclerosis, alongside examining the correlation between liver fibrosis predicted by non-invasive indicators and coronary heart disease. Additionally, the research delves into the mechanisms underlying inflammation.

## Methods

### Study population

**Fig. 1:**
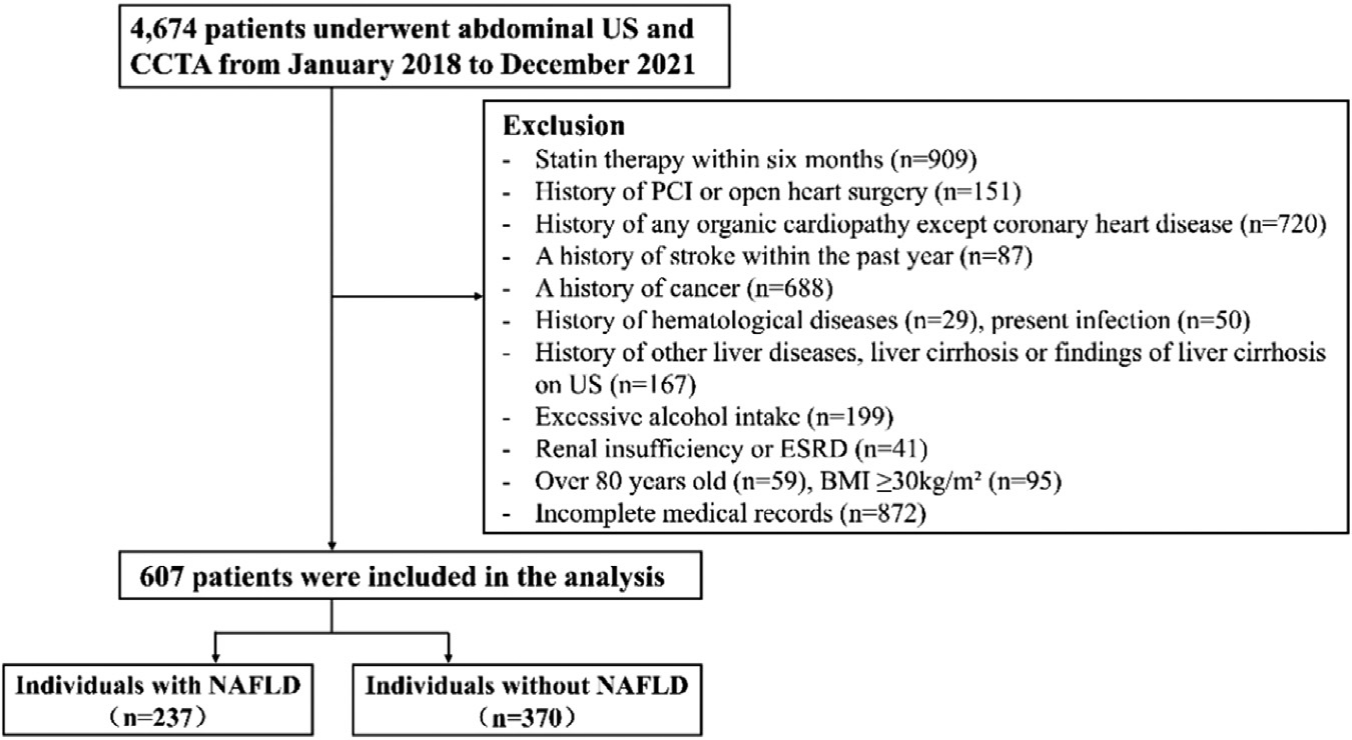
The flowchart of the study population. CCTA, Coronary Computed Tomography Angiography; PCI, Percutaneous Coronary Intervention; US, Ultrasonography; ESRD, End-Stage Renal Disease; BMI, Body Mass Index.

The authors continuously collected the clinical data of 4674 patients who underwent coronary computed tomography angiography (CCTA) and abdominal ultrasonography (US) in the First Affiliated Hospital of Chongqing Medical University from January 2018 to December 2021 and conducted a retrospective cross-sectional study on this part of the population (Fig. 1). According to the following criteria, 4067 patients were excluded: i) Received statin therapy within half a year; ii) History of open heart surgery or percutaneous coronary intervention (PCI); iii) Any history of organic heart disease except coronary heart disease, cerebrovascular accident history in the past year, cancer history, blood system disease, current infection; iv) Other liver diseases (such as viral hepatitis or autoimmune liver disease), history of cirrhosis or liver cirrhosis found by ultrasonography; v) Excessive drinking (male ≥ 30 g/day, female ≥ 20 g/day); vi) Renal insufficiency or end-stage renal disease (ESRD); vii) Age ≥80 years, body mass index (BMI) ≥ 30 kg/m^2^; viii) Incomplete medical record information. Finally, 607 patients were included in the study. This study followed the STROBE Statement.

### Clinical and laboratory data

Basic demographic data, such as age, gender, height, weight, blood pressure, smoking, drinking, and family history of CVD were collected. Disease history included hypertension, diabetes, heart failure, myocardial infarction, cerebrovascular accident, cancer, etc. Surgical history included open heart surgery and PCI. Medical history data were derived from electronic medical records. Laboratory data were also measured on admission, including neutrophil count, lymphocyte count, monocyte count, platelet count, total cholesterol (TC), triglyceride (TG), low-density lipoprotein cholesterol (LDL-C), high-density lipoprotein cholesterol (HDL-C), hypersensitive C-reactive protein (hs-CRP), albumin, total bilirubin (TBIL), direct bilirubin (DBIL), aspartate transaminase (AST), alanine transaminase (ALT), γ-glutamyl transpeptidase (GGT), urea, creatinine, uric acid, fasting blood glucose (FBG), and glycated hemoglobin (HbA1c). The presence of fatty liver was determined by ultrasonography examination and the presence of carotid intima-media thickening was determined by carotid vascular ultrasound.

The body mass index was calculated as weight in kilograms divided by the square of height in meters (kg/m^2^). Obesity is defined as BMI ≥ 30 kg/m^2^.^8^ Hypertension was defined as systolic blood pressure ≥ 140 mmHg and/or diastolic blood pressure ≥ 90 mmHg, or a history of hypertension. Diabetes was defined as fasting blood glucose ≥ 7.0 mmoL/L or HbA1c ≥ 6.5 %, or a history of diabetes.

### Noninvasive inflammatory markers and fibrosis markers

In addition to hs-CRP, the authors also used systemic inflammatory indexes to assess the systemic inflammatory status in patients with NAFLD, and the formulas for these indicators are as follows: NLR, Neutrophil-to-Lymphocyte Ratio; PLR, Platelet-to-Lymphocyte Ratio; LMR, Lymphocyte-to-Monocyte Ratio; MHR, Monocyte-to-HDL-C Ratio; Systemic Immune Inflammation Index (SII), NLR* platelet count; Systemic Inflammatory Response Index (SIRI), NLR*monocyte count.^9^^,^^10^

To predict the state of liver fibrosis in patients with NAFLD, the authors calculated the Fibrosis-4 (FIB-4) index, aspartate Aminotransferase, and Platelet Ratio Index (APRI) score. These fibrosis markers were calculated according to the following formulas: FIB-4 = (age (years) × AST (U/L)) / (platelet count (× 10^9 /L) × ALT (U/L)^1/2), the low and high probability of advanced fibrosis critical index were 1.3 and 2.67; APRI = 100 × (AST / upper limit of normal) / platelet count (× 10^9 /L), the cut-off values of low and high incidence of advanced fibrosis were 0.5 and 1.5, respectively.^11^

### Coronary computed tomography angiography

CCTA was used to assess the degree of coronary atherosclerosis. The coronary artery calcium score (CACS) was measured and categorized by scores of 0, 1 to 10, 11 to 100, 101 to 400, and >400. Coronary artery calcification was defined as CACS > 10.^12^ Plaques with calcified tissue accounting for more than 50 % of the plaque area (density >130 HU) were classified as calcified plaques, plaques with calcium content < 50 % were classified as mixed plaques, and plaques without calcium were classified as non-calcified plaques. CHD is defined as the presence of at least one major coronary artery and its large branch vessel diameter stenosis ≥ 50 %.

### Statistical analysis

Statistical analyses were performed using IBM SPSS Statistics v. 26.0 (SPSS Inc., Chicago, Illinois, USA). Continuous variables are expressed as mean ± standard deviation or median (IQR), and categorical variables are expressed as the number of cases and percentages. In the univariate analysis, the unpaired *t*-test or Mann-Whitney test was used to compare continuous variables, and the Chi-Square test or Fisher's exact test was used to compare categorical variables. The binary logistic regression model was used to analyze the relationship between NAFLD and atherosclerosis, and the correlation between liver fibrosis and atherosclerosis. The covariates in the multivariate regression model were selected according to their clinical importance and statistical significance. The Receiver Operating Characteristic (ROC) curves were constructed to evaluate the predictive value of fibrosis markers and inflammatory markers for CHD. Mediation analysis was performed by the PROCESS macro (Process V4.1) developed by Hayes and Preacher in SPSS version 26.0 software.^13^ PROCESS macro program is a plug-in program based on SPSS and SAS for conducting mediation and moderating effects analysis, which refers to the idea of regression analysis, and can analyze various mediation models, moderating models, and combined models among them, and is characterized by easy operation and comprehensive presentation of results. Figure S1 is a mediation analysis path diagram. All reported p values were bilateral, and *p* < 0.05 was considered statistically significant.

## Results

### Baseline characteristics

As shown in Table 1, the average age of the study population was 62.4 ± 10.3 years, and 210 (34.6 %) patients were male. Among the included patients, 237 (39.0 %) were diagnosed with NAFLD via ultrasound. Compared with the patients without NAFLD, the former group exhibited a higher proportion of males and higher smoking rates, a greater prevalence of family history of CVD, diabetes and hypertension, elevated BMI, uric acid, ALT, AST, TG, hs-CRP, and APRI levels, as well as a lower HDL-C level. However, no significant differences were observed in age, creatinine, TC, LDL-C, and FIB-4 between the two groups.

**Table 1:** Baseline characteristics of individuals with and without NAFLD.

Characteristics	Overall (*n* = 607)	Non-NAFLD (*n* = 370)	NAFLD (*n* = 237)	p-value
Age (year), mean ± SD	62.4 ± 10.3	62.8 ± 10.8	61.8 ± 9.4	0.264
Male, n (%)	210 (34.6)	114 (30.8)	96 (40.5)	0.014
Smoking, n (%)	129 (21.3)	66 (17.8)	63 (26.6)	0.010
Family history of CVD, n (%)	174 (28.7)	86 (23.2)	88 (37.1)	<0.001
Diabetes mellitus, n (%)	180 (29.7)	70 (18.9)	110 (46.4)	<0.001
Hypertension, n (%)	337 (55.5)	190 (51.4)	147 (62.0)	<0.010
Systolic blood pressure (mmHg), mean ± SD	133.0 ± 18.6	131.9 ± 18.7	134.8 ± 18.2	0.065
Diastolic blood pressure (mmHg), mean ± SD	79.3 ± 11.9	78.1 ± 12.1	81.1 ± 11.5	0.002
BMI (kg/m^2^), mean ± SD	24.1 ± 2.8	23.1 ± 2.8	25.5 ± 2.2	<0.001
Urea (mmol/L), mean ± SD	5.7 ± 3.0	5.7 ± 3.6	5.7 ± 1.6	0.989
Creatinine (μmoL/L), mean ± SD	68.1 ± 23.7	66.9 ± 18.5	69.9 ± 30.0	0.126
Uric acid (μmoL/L), mean ± SD	232.7 ± 81.0	300.9 ± 73.2	333.8 ± 88.3	<0.001
Albumin (g/L), mean ± SD	43.5 ± 3.9	43.4 ± 4.1	43.6 ± 3.5	0.630
TBIL (μmoL/L), median (IQR)	9.5 (5.5)	9.7 (5.6)	9.3 (4.9)	0.822
DBIL (μmoL/L), median (IQR)	4.0 ± 1.8	4.0 ± 1.9	4.0 ± 1.7	0.696
ALT (U/L), median (IQR)	18 (13)	16 (11)	22 (15)	<0.001
AST (U/L), median (IQR)	19 (7)	18 (8)	20 (9)	0.001
GGT (U/L), median (IQR)	21 (17)	18 (13)	26 (17)	<0.001
Total cholesterol (mmoL/L), median (IQR)	1.4 (1.0)	1.2 (0.7)	1.7 (1.2)	<0.001
Triglyceride (mmoL/L), mean ± SD	4.7 ± 1.0	4.7 ± 0.9	4.6 ± 1.0	0.500
HDL-C (mmoL/L), mean ± SD	1.3 ± 0.4	1.4 ± 0.4	1.1 ± 0.3	<0.001
LDL-C (mmoL/L), mean ± SD	2.9 ± 0.9	2.9 ± 0.8	2.9 ± 0.9	0.922
Hs-CRP (mg/L), median (IQR)	0.9 (1.4)	0.7 (1.1)	1.2 (1.7)	<0.001
HbA1C (%), median (IQR)	5.8 (0.7)	5.7 (0.5)	6.1 (1.4)	<0.001
FBG (mmoL/L), median (IQR)	5.5 (1.2)	5.3 (0.9)	5.8 (2.2)	<0.001
Fibrosis markers				
FIB-4, median (IQR)	1.3 (0.8)	1.4 (0.7)	1.3 (0.7)	0.078
APRI, median (IQR)	0.27 (0.15)	0.27 (0.16)	0.27 (0.14)	0.002

Values are expressed as mean ± standard deviation, number (%), or median (interquartile range). BMI, Body Mass Index; TBIL, Total Bilirubin; DBIL, Direct Bilirubin; ALT, Alanine Aminotransferase; AST, Aspartate Aminotransferase; GGT, γ-Glutamyl Transpeptidase; HDL-C, High-Density Lipoprotein Cholesterol; LDL-C, Low-Density Lipoprotein Cholesterol; hs-CRP, Hypersensitive C-Reactive Protein; FBG, Fasting Blood Glucose.

### NAFLD and atherosclerosis

The difference analysis of atherosclerosis characteristics between patients with and without NAFLD is shown in Table 2. The prevalence of carotid intima thickening, coronary atherosclerotic plaque, calcified plaque, and non-calcified plaque was significantly higher among individuals with NAFLD. However, no significant difference was observed in mixed plaque (*p* = 0.256). Among the participants, 99 (16.3 %) patients were diagnosed with coronary artery disease through CCTA. Notably, the prevalence of coronary heart disease was significantly greater in individuals with NAFLD when compared to those without NAFLD (*p* = 0.003). Furthermore, the median CACS for the entire study population was 0.0 (17.5). The CACS of individuals with NAFLD was found to be higher as opposed to those without NAFLD (*p* = 0.033). Moreover, a notable disparity existed in the distribution of CACS classification between the two groups (*p* = 0.038).

**Table 2:** Comparison of atherosclerosis characteristics between patients with and without NAFLD.

Variables	Overall (*n* = 607)	Non-NAFLD (*n* = 370)	NAFLD (*n* = 237)	p-value
Carotid intima thickening, n (%)	334 (55.0)	187 (50.5)	147 (62.0)	0.006
Coronary atherosclerotic plaque, n (%)	278 (45.8)	153 (41.4)	125 (52.7)	0.006
Plaque type, n (%)				
Calcified plaque	188 (31.0)	100 (27.0)	88 (37.1)	0.009
Non-calcified plaque	178 (29.3)	92 (24.9)	86 (36.3)	0.003
Mixed plaque	47 (7.7)	25 (6.8)	22 (9.3)	0.256
CHD, n (%)	99 (16.3)	47 (12.7)	52 (21.9)	0.003
CACS, median (IQR)	0.0 (17.5)	0.0 (11.1)	0.0 (29.2)	0.033
CACS classification, n (%)				0.038
0	401 (66.1)	258 (69.7)	143 (60.3)	
1‒10	43 (7.1)	20 (5.4)	23 (9.7)	
11‒100	82 (13.5)	47 (12.7)	35 (14.8)	
101‒400	55 (9.1)	27 (7.3)	28 (11.8)	
>400	26 (4.3)	18 (4.9)	8 (3.4)	

Values are expressed as number (%) or median (interquartile range). CACS, Coronary Artery Calcium Score; CHD, Coronary Artery Disease.

The binary logistic regression analysis of NAFLD and atherosclerosis is shown in Table S1. Upon adjusting for traditional cardiovascular risk factors (age, gender, smoking, family history of CVD, diabetes, hypertension, TG, HDL-C, LDL-C), as well as ALT and AST, logistic regression analysis indicated a continued significant correlation between NAFLD and carotid intima thickening (1.58, 95 % CI 1.04‒2.40; *p* = 0.034), as well as non-calcified plaque (1.56, 95 % CI 1.03‒2.37; *p* = 0.038). Conversely, NAFLD did not show an associated with coronary atherosclerotic plaque (1.33, 95 % CI 0.89‒1.99; *p* = 0.168), calcified plaque (1.36, 95 % CI 0.89‒2.08; *p* = 0.162), mixed plaque (0.90, 95 % CI 0.44‒1.84; *p* = 0.766), coronary heart disease (1.50, 95 % CI 0.88‒2.53; *p* = 0.134), or coronary artery calcification (1.04, 95 % CI 0.66‒1.62; *p* = 0.878).

### The relationship between NAFLD fibrosis markers and atherosclerosis

**Fig. 2:**
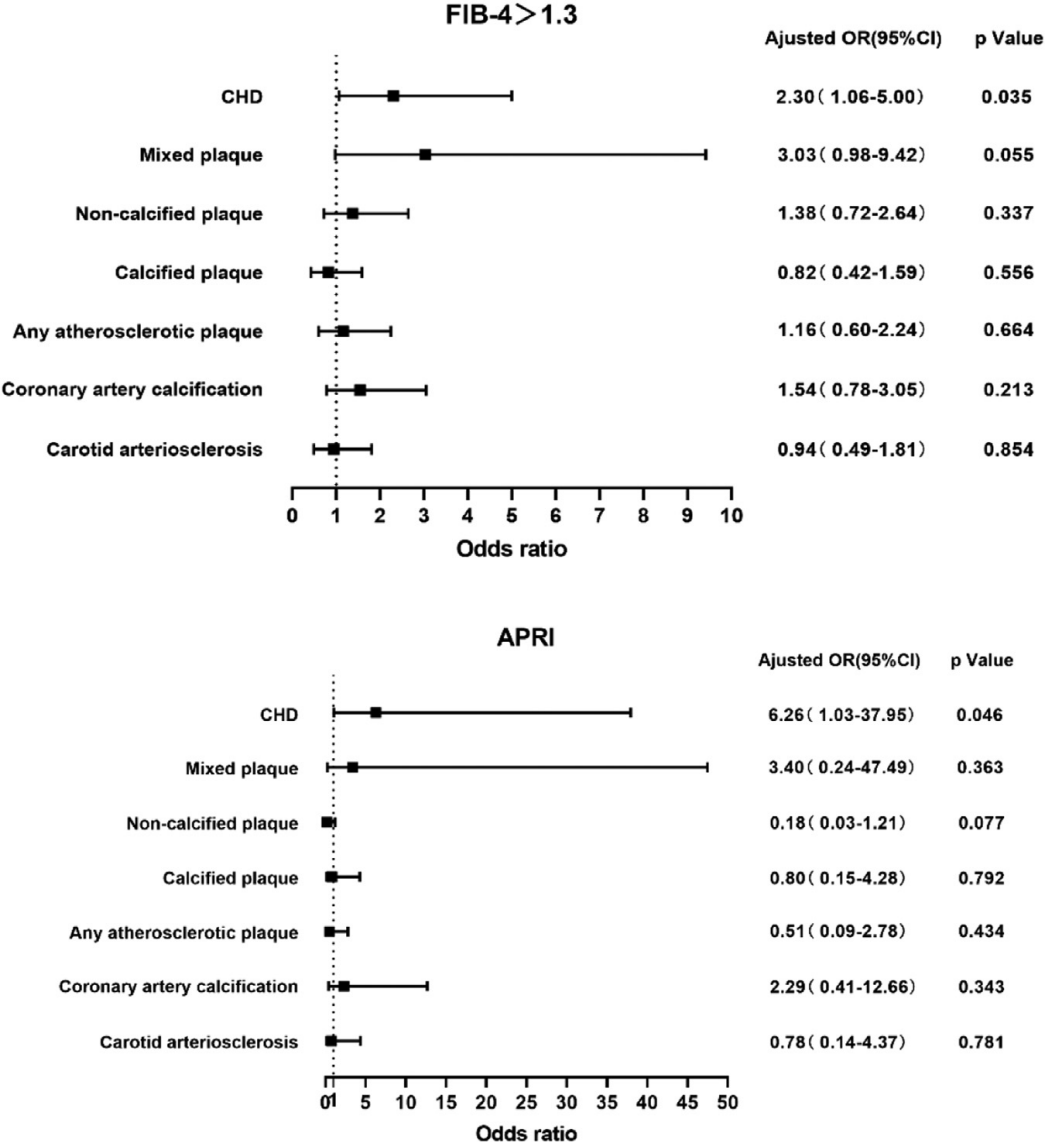
A forest map was created based on a multivariate logistic regression model of fibrosis markers and atherosclerosis. This diagram displays the odds ratio of FIB-4 and APRI in relation to atherosclerosis after adjusting for various traditional cardiovascular risk factors.

Fig. 2 presents the forest plot illustrating the relationship between atherosclerosis and NAFLD fibrosis as predicted by FIB-4 and APRI. Upon adjusting for traditional cardiovascular risk factors (age, gender, smoking, family history of CVD, diabetes mellitus, hypertension, TG, HDL-C, LDL-C), FIB-4 >1.3 demonstrated a significant association with CHD (2.30, 95 % CI 1.06‒5.00; *p* = 0.035). However, no significant correlations were observed with carotid intima thickening, coronary artery calcification, coronary atherosclerotic plaque, calcified plaque, non-calcified plaque, or mixed plaque. Similarly, APRI was found to be independently associated only with CHD (6.26, 95 % CI 1.03‒37.95; *p* = 0.046).

### Relationship between inflammatory markers and CHD

Table 3 illustrates the relationship between inflammatory markers and CHD. Among the markers considered, including hs-CRP and systemic inflammatory indexes, the results of univariate regression indicated significant associations between NLR, LMR, and SIRI with CHD. However, upon adjusting for traditional cardiovascular risk factors (age, gender, smoking, family history of CVD, diabetes mellitus, hypertension, TG, HDL-C, LDL-C), the findings of multivariate regression demonstrated that only NLR (1.56, 95 % CI 1.08‒2.26; *p* = 0.018) and SIRI (1.95, 95 % CI 1.16‒3.27; *p* = 0.012) retained their independent associations with the combination of NAFLD and CHD.

**Table 3:** Association between inflammation and CHD in the binary logistic regression model.

Variables	Univariate		Multivariate	
	Odds ratio (95 % CI)	p-value	Odds ratio (95 % CI)	p-value
hs-CRP	0.99 (0.89‒1.09)	0.814	0.96 (0.85‒1.09)	0.538
NLR	1.81 (1.31‒2.50)	<0.001	1.56 (1.08‒2.26)	0.018
PLR	1.01 (0.99‒1.02)	0.320	1.01 (1.00‒1.03)	0.197
LMR	0.90 (0.77‒1.04)	0.015	0.92 (0.78‒1.10)	0.361
MHR	3.23 (0.78‒13.50)	0.107	3.94 (0.45‒34.42)	0.215
SII	1.00 (1.00‒1.01)	0.119	1.00 (0.99‒1.00)	0.477
SIRI	2.29 (1.45‒3.60)	<0.001	1.95 (1.16‒3.27)	0.012

Univariate and multifactorial analyses were performed using logistic regression models. The covariates in the multifactorial regression model included age, sex, smoking, family history of CVD, diabetes mellitus, hypertension, TG, HDL-C, and LDL-C.

### ROC curve analysis of fibrosis markers and inflammation markers

**Fig. 3:**
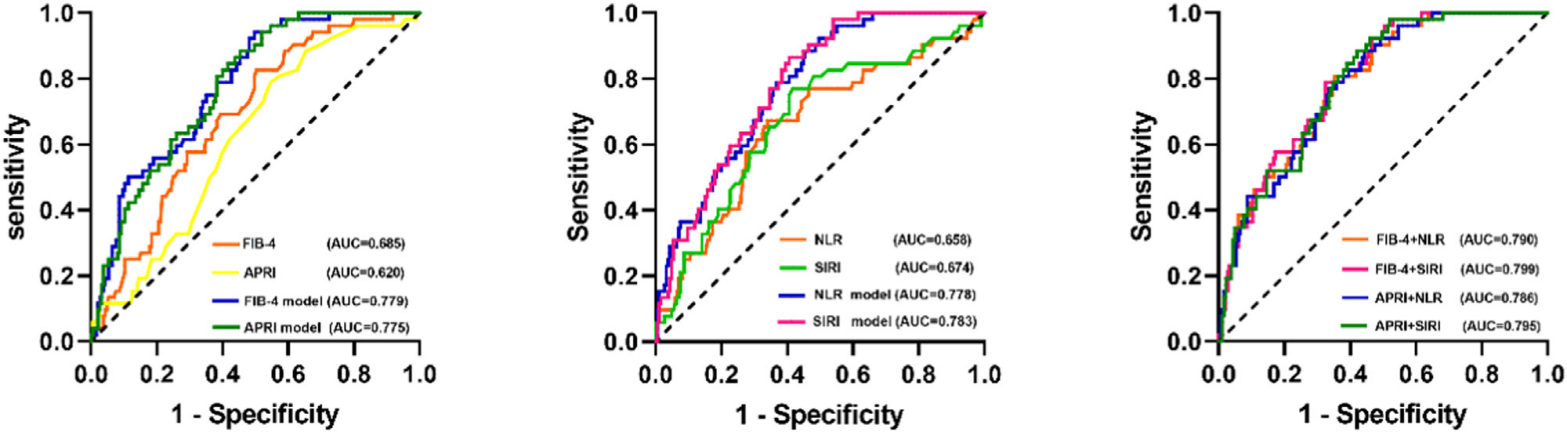
The Receiver Operating Characteristic (ROC) curve illustrates the diagnostic efficacy of fibrosis markers and systemic inflammation indexes for CHD. The FIB-4 model, APRI model, PLR model, FIB-4+PLR, and APRI+PLR represent novel prediction models that integrate traditional cardiovascular risk factors (age, gender, smoking, family history of CVD, diabetes, hypertension, TG, HDL-C, LDL-C) using non-invasive markers.

The results of the ROC curve (Fig. 3) showed that the Area Under the Receiver Operating Characteristic Curves (AUROCs) for FIB-4, APRI, NLR, and SIRI to be 0.685 (0.610‒0.759), 0.620 (0.541‒0.699), 0.658 (0.572‒0.745), and 0.674 (0.590‒0.759), respectively. Upon integration of traditional cardiovascular risk factors, the AUROCs for these models exhibited an increase compared to the original model, resulting in values of 0.779, 0.775, 0.778, and 0.783, respectively. When combining fibrosis markers with systemic inflammatory indexes and traditional cardiovascular risk factors, AUROCs increased slightly, which were 0.790, 0.799, 0.786 and 0.795, respectively.

### Mediating analysis of the correlation between NAFLD fibrosis markers and CHD

**Fig. 4:**
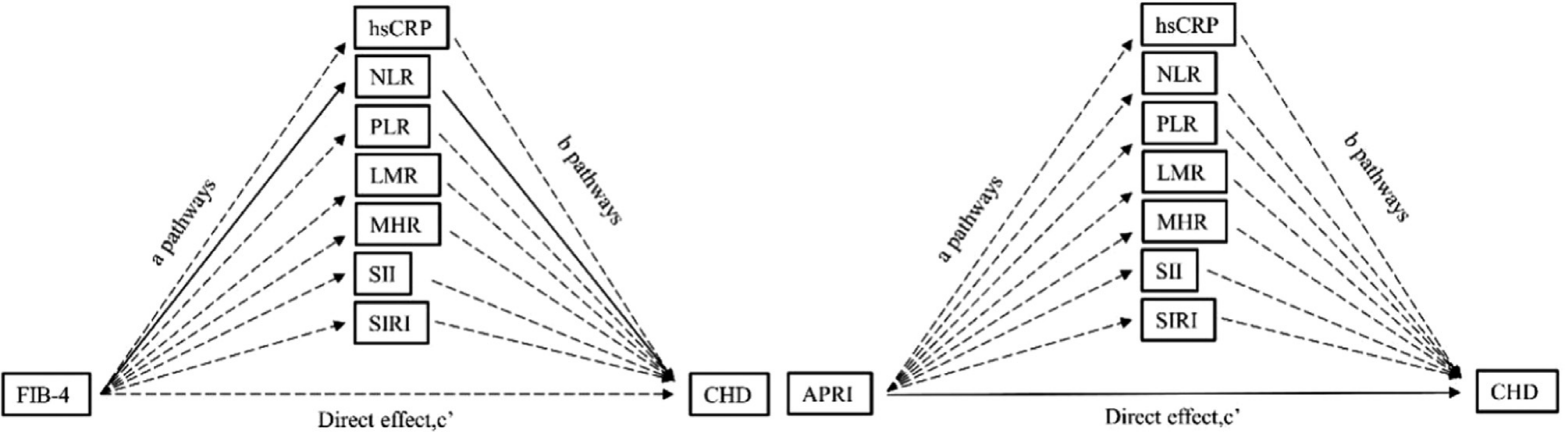
The mediating effect model illustrating the impact of inflammation-mediated fibrosis markers on CHD is depicted. The coefficient *a* is the effect of fibrosis markers on inflammatory markers; the coefficient *b* is the effect of inflammatory markers on CHD after controlling for the influence of fibrosis markers; the coefficient *c’* is the direct effect of fibrosis markers on CHD after controlling for the influence of inflammatory markers. The solid line indicates that the path is statistically significant.

The outcomes of the mediating analysis concerning the impact of inflammation-mediated NAFLD fibrosis on coronary heart disease are presented in Table S2. Within the FIB-4 mediated model, solely the 95 % Confidence Interval of NLR (with a mediating effect value of 0.068, 95 % CI 0.003 < 0.232) does not contain zero, indicating a statistically significant mediating effect. Furthermore, with the mediating variable NLR controlled, the influence of FIB-4 on coronary heart disease demonstrated insignificance (i.e., the direct effect was not significant). Within the APRI-mediated model, the 95 % Confidence Intervals for hs-CRP and systemic inflammatory indexes contained zero, signifying an absence of significant mediating effects. Fig. 4 outlines the pathway of the aforementioned mediating effect model. The effect of FIB-4 on coronary heart disease is indirectly mediated via the NLR-mediated a*b pathway, while the *c'* pathway exhibits no direct effect.

## Discussion

The primary outcomes of this study revealed that NAFLD was an independent risk factor for carotid intima thickening and non-calcified plaque. Moreover, NAFLD fibrosis, as predicted by the FIB-4 index and the APRI score, demonstrated an independent correlation with the occurrence of CHD. In the early stages of NAFLD, systemic inflammation might perform a comprehensive mediating function in the connection between liver fibrosis and coronary heart disease.

Increasing evidence suggests that NAFLD may also be an independent risk factor for CVD morbidity and mortality in addition to traditional cardiovascular risk factors such as age, smoking and diabetes.^5^^,^^14^^,^^15^ However, a community-based cohort follow-up study showed that NAFLD might not be indicative of 10-year all-cause mortality and cardiovascular mortality.^16^ Presently, coronary heart disease continues to be the most prevalent cardiovascular ailment, carrying the highest economic burden. Prior research has established a connection between NAFLD featuring advanced fibrosis and cardiovascular mortality.^17^ Nevertheless, only a limited number of studies have disclosed a close link between NAFLD fibrosis and the severity and prognosis of CHD.^18^, ^19^, ^20^ Additionally, these studies have not particularly concentrated on NAFLD and atherosclerosis in their early stages. In clinical practice, the application of non-invasive fibrosis scoring systems for early detection and assessment of CVD risk in NAFLD patients bears considerable clinical significance. Therefore, the objective of this study is to assess the association between early-stage NAFLD, its fibrosis extent, and atherosclerosis, while also delving into the potential mediating role of inflammation.

In this study, CCTA was used to describe the type of atherosclerotic plaque and determine the presence of coronary artery calcification and stenosis. The present findings revealed that 237 (39.0 %) patients received a diagnosis of NAFLD via ultrasonography, a result consistent with previous research outcomes.^21^^,^^22^ Following adjustment for traditional cardiovascular risk factors, ALT, and AST, the study showed that NAFLD exhibited no connection with coronary artery calcification, calcified plaque, mixed plaque, or CHD. However, it was associated solely with carotid intima thickening and non-calcified plaque. Research has indicated a link between NAFLD and both coronary artery calcification and its progression.^22^^,^^23^ Yet, Lee et al.^21^ reported an absence of significant associations between NAFLD and coronary artery calcification, calcified plaque, or mixed plaque. Nevertheless, they consistently found an independent association with non-calcified plaque, aligning with the present findings. Earlier studies have highlighted that high-risk plaque types like non-calcified plaque significantly elevate the likelihood of future acute coronary syndrome occurrences.^24^^,^^25^ Furthermore, patients with NAFLD exhibit a higher prevalence of high-risk plaques compared to those without NAFLD,^26^ a trend corroborated by this study. Consequently, the elevated risk of plaque rupture might play a partial role in the mechanism underpinning adverse cardiovascular events in NAFLD patients.

Furthermore, the present study adopted a non-invasive fibrosis scoring system to anticipate the extent of liver fibrosis. Compared with the invasive and limitations of liver biopsy, the non-invasive fibrosis scoring system offers advantages such as affordability, operational ease, and widespread applicability, making it the chosen methodology in this study. Existing research has highlighted the potential of FIB-4, APRI, and NFS in predicting advanced fibrosis as well as the stratification of liver-related morbidity and mortality risks in NAFLD patients. Among these, FIB-4 and NFS have demonstrated superior diagnostic efficacy.^11^^,^^27^ This investigation discovered that post-adjustment for traditional cardiovascular risk factors, FIB-4 >1.3 and APRI displayed independent associations with the onset of CHD. This implies that individuals exhibiting early-stage liver fibrosis in the context of NAFLD are more prone to the development of coronary heart disease. Furthermore, ROC analysis revealed that the incorporation of traditional cardiovascular risk factors alongside fibrosis markers or inflammatory markers significantly enhanced the predictive capacity for CHD. Notably, the incorporation of both markers showed the highest predictive capability for CHD. Similar conclusions were drawn by Chen et al.^20^ in their research. Hence, for NAFLD patients, the utilization of non-invasive fibrosis scoring systems like the FIB-4 index and the APRI score holds clinical significance in anticipating the emergence of CHD. This practice aids in the early identification of high-risk individuals within the NAFLD population.

The liver harbors a significant population of macrophages and immune cells. When simple fatty liver transitions to NASH, the release of inflammatory cytokines by these entities into the bloodstream can incite systemic inflammation. This phenomenon not only assumes a pivotal role in the histological advancement of NAFLD^28^ but also partly fuels the progression of atherosclerosis.^29^ In the present context, evidence indicates a correlation between parameters of white blood cells and their subtype ratios with escalated cardiovascular risk and mortality.^30^^,^^31^ As novel inflammatory markers, NLR and SIRI are closely related to the severity of coronary artery disease.^32^^,^^33^ The present study also confirmed that NLR and SIRI are independent risk factors for CHD. Indeed, neutrophils and their derivatives can accelerate atherosclerosis and propel plaque progression by instigating inflammation, recruiting monocytes, inducing oxidative stress, and prompting foam cell formation. The presence of neutrophils within the lesion is additionally associated with plaque instability, attributed to protease expression and a predisposition toward bleeding.^34^ The exact role of lymphocyte reduction in atherosclerosis formation remains not fully elucidated. While innate-like B1 B-cells confer a protective influence against atherosclerosis, adaptive B2 B-cells, through interaction with other leukocytes and/or the secretion of inflammatory cytokines, foster disease progression.^35^ Consequently, within the early-stage NAFLD population, the higher the systemic inflammation level indicated by systemic inflammatory indexes, the greater the susceptibility to developing coronary heart disease.

It is noteworthy that the mechanism underlying the heightened atherosclerosis risk in the context of NAFLD remains incompletely understood. Inflammation, as a pivotal intermediary, might not only act as a shared risk factor for both conditions but also potentially function as a connecting link between them. Given this, the present study adopted mediation analysis to delve into the mediating role of inflammation between liver fibrosis and CHD within the early-stage NAFLD population. Notably, mediation analysis was a method commonly utilized in psychological and sociological research in the past, with limited application in other domains. However, its use within the cardiovascular sphere has been progressively expanding in recent years.^36^^,^^37^ The results show that the mediating effect of FIB-4 mediated by NLR on coronary heart disease was significant, and the direct effect was not significant, which means that within the early-stage NAFLD population, liver fibrosis might primarily spur atherosclerosis progression through the conduit of inflammation. In contrast, Chen et al.’s^20^ mediation analysis indicated both mediating and direct effects concerning the association between NAFLD fibrosis markers and the coronary Gensini score. The disparity in the present results could be attributed to the predominant inclusion of early to intermediate fibrosis patients within the NAFLD cohort, with a limited representation of advanced fibrosis cases. These findings emphasize the importance of prevention to avoid cardiovascular events in asymptomatic NAFLD patients. For this population, early screening, and assessment of non-invasive fibrosis markers, coupled with timely lifestyle interventions and appropriate pharmacotherapy if warranted, could potentially enhance the prognosis of NAFLD patients.

The present study does come with certain limitations. Firstly, it was a retrospective single-center investigation, encompassing a relatively modest patient sample. Additionally, the research lacked extensive follow-up data, thereby impeding the capacity to establish a causative role of liver fibrosis in atherosclerosis progression or undertake prognostic analyses. Therefore, these findings need to be further confirmed in more prospective follow-up studies. Secondly, the study defined the presence of fatty liver based on ultrasound findings, which are less sensitive (60 %‒90 %) when hepatic fatty infiltration is below approximately 30 %.^38^ Although computed tomography offers heightened accuracy in diagnosing fatty liver in clinical contexts, ultrasound remains a widely employed method in both clinical settings and population-based studies owing to its attributes of radiation-free assessment and cost-effectiveness. Thirdly, the employment of CCTA for plaque-type and stenosis degree analysis, while valuable, may present a marginally diminished clarity and precision when contrasted with coronary angiography. This could potentially lead to an underrepresentation of patients with severe CHD stenosis, given that a majority of such patients undergo direct coronary angiography. Hence, the present study could bear a degree of selection bias. In summation, while the mediation effect analysis is a preliminary endeavor to explore the plausible causal connection between early-stage NAFLD fibrosis and CHD, it is insufficient to elucidate the underlying pathophysiological mechanisms. Thus, subsequent research efforts are imperative.

## Conclusion

In conclusion, the cross-sectional study involving individuals with early-stage NAFLD has uncovered associations between NAFLD and carotid intimal thickening, as well as non-calcified plaque ‒ highlighting an elevated cardiovascular risk within this population. Furthermore, we've established NAFLD fibrosis as an independent CHD risk factor, with inflammation potentially serving as a complete mediator in the connection between liver fibrosis and CHD. As a result, proactive early screening, and intervention in individuals with early-stage NAFLD remain imperative to forestall the evolution of cardiovascular disease and subsequent major adverse cardiovascular events.

## Ethics approval and consent to participate

The study was approved by the Ethics Committee of the First Affiliated Hospital of Chongqing Medical University (approval no. K2023–098) and was conducted in accordance with the 1964 Helsinki Declaration and its later amendments or comparable ethical standards. Because the research only retrospectively accessed a de-identified database for purposes of analyses, the informed consent requirement was exempted by the ethics committee.

## Authors' contributions

Si-hua Tan was involved in the study concept and design, data collection, data analysis, data interpretation and manuscript writing.

Xiao-li Zhou was involved in the study concept and design and modify the manuscript. All authors have approved the final draft.

## Financial

This research did not receive any specific grant from funding agencies in the public, commercial, or not-for-profit sectors.

## Declaration of Competing Interest

The authors declare no conflicts of interest.

## Appendix Supplementary materials

Image, application 1
