# Supplementary material for: Early-stage non-alcoholic fatty liver disease in relation to atherosclerosis and inflammation
Source: Clinics (Sao Paulo). 2023 Nov 11;78:100301. doi: 10.1016/j.clinsp.2023.100301 (PMC10681951; doi:10.1016/j.clinsp.2023.100301)

CLINICS-D-23-00275_Supplementary Material

**Table S1** Association between NAFLD and atherosclerosis in a logistic multifactorial regression model.

| **Variables** | **Multivariate** | |
| --- | --- | --- |
|  | **Odds ratio (95% CI)** | **p-value** |
| Carotid intima thickening |  |  |
| Normal (reference) | 1 | ‒ |
| NAFLD | 1.58 (1.04‒2.40) | 0.034 |
| Coronary atherosclerotic plaque |  |  |
| Normal (reference) | 1 | ‒ |
| NAFLD | 1.33 (0.89‒1.99) | 0.168 |
| Calcified plaque |  |  |
| Normal (reference) | 1 | ‒ |
| NAFLD | 1.36 (0.89‒2.08) | 0.162 |
| Non-calcified plaque |  |  |
| Normal (reference) | 1 | ‒ |
| NAFLD | 1.56 (1.03‒2.37) | 0.038 |
| Mixed plaque |  |  |
| Normal (reference) | 1 | ‒ |
| NAFLD | 0.90 (0.44‒1.84) | 0.766 |
| Coronary artery disease |  |  |
| Normal (reference) | 1 | ‒ |
| NAFLD | 1.50 (0.88‒2.53) | 0.134 |
| Coronary artery calcification |  |  |
| Normal (reference) | 1 | ‒ |
| NAFLD | 1.04 (0.66‒1.62) | 0.878 |

Multivariate analysis was performed using a logistic regression model. Covariates included age, gender, smoking, family history of CVD, diabetes mellitus, hypertension, TG, HDL-C, LDL-C, ALT, and AST. Coronary artery calcification was defined as CACS >10.

**Table S2** Inflammatory markers mediating the association between NAFLD fibrosis and CHD.

| (A) FIB-4 predicts liver fibrosis | | | | | | | | | | | | | |
| --- | --- | --- | --- | --- | --- | --- | --- | --- | --- | --- | --- | --- | --- |
| **Inflam-matory markers** |  | | | | |  | | | **Indirect effect** | | | | |
|  |  | **β** | **SE** | **P值** |  | **β** | **SE** | **P值** |  | **β** | **SE** | **Boot LLCI** | **Boot ULCI** |
| hs-CRP | *a* | 0.272 | 0.269 | 0.313 | *c’* | 0.486 | 0.253 | 0.055 | *a*b* | -0.013 | 0.062 | -0.196 | 0.055 |
|  |  |  |  |  | *b* | -0.049 | 0.063 | 0.433 |  |  |  |  |  |
| NLR | *a* | 0.173 | 0.076 | 0.023 | *c’* | 0.400 | 0.237 | 0.091 | *a*b* | 0.068 | 0.058 | 0.003 | 0.232 |
|  |  |  |  |  | *b* | 0.395 | 0.190 | 0.037 |  |  |  |  |  |
| PLR | *a* | -2.925 | 1.977 | 0.140 | *c’* | 0.499 | 0.258 | 0.053 | *a*b* | -0.035 | 0.080 | -0.290 | 0.012 |
|  |  |  |  |  | *b* | 0.012 | 0.008 | 0.140 |  |  |  |  |  |
| LMR | *a* | -0.399 | 0.192 | 0.039 | *c’* | 0.441 | 0.232 | 0.058 | *a*b* | 0.026 | 0.073 | -0.045 | 0.253 |
|  |  |  |  |  | *b* | -0.066 | 0.089 | 0.459 |  |  |  |  |  |
| MHR | *a* | -0.021 | 0.013 | 0.121 | *c’* | 0.498 | 0.252 | 0.049 | *a*b* | -0.035 | 0.069 | -0.247 | 0.019 |
|  |  |  |  |  | *b* | 1.668 | 1.142 | 0.144 |  |  |  |  |  |
| SII | *a* | -36.366 | 15.938 | 0.023 | *c’* | 0.504 | 0.261 | 0.054 | *a*b* | -0.034 | 0.088 | -0.304 | 0.060 |
|  |  |  |  |  | *b* | 0.001 | 0.001 | 0.310 |  |  |  |  |  |
| SIRI | *a* | 0.034 | 0.053 | 0.530 | *c’* | 0.445 | 0.246 | 0.071 | *a*b* | 0.022 | 0.039 | -0.066 | 0.093 |
|  |  |  |  |  | *b* | 0.648 | 0.268 | 0.016 |  |  |  |  |  |
| (B) APRI predicts liver fibrosis. | | | | | | | | | | | | | |
| **Inflam-matory markers** |  | | | |  | | | | **Indirect effect** | | | | |
|  |  | **β** | **SE** | **P值** |  | **β** | **SE** | **P值** |  | **β** | **SE** | **Boot LLCI** | **Boot ULCI** |
| hs-CRP | *a* | -0.461 | 1.209 | 0.703 | *c’* | 1.809 | 0.922 | 0.049 | *a*b* | 0.015 | 0.102 | -0.108 | 0.301 |
|  |  |  |  |  | *b* | -0.032 | 0.063 | 0.611 |  |  |  |  |  |
| NLR | *a* | 0.589 | 0.340 | 0.085 | *c’* | 1.710 | 0.946 | 0.071 | *a*b* | 0.245 | 0.220 | -0.065 | 0.789 |
|  |  |  |  |  | *b* | 0.416 | 0.187 | 0.026 |  |  |  |  |  |
| PLR | *a* | -21.597 | 8.779 | 0.015 | *c’* | 2.088 | 0.952 | 0.028 | *a*b* | -0.289 | 0.227 | -0.863 | 0.026 |
|  |  |  |  |  | *b* | 0.013 | 0.008 | 0.104 |  |  |  |  |  |
| LMR | *a* | -2.480 | 0.854 | 0.004 | *c’* | 1.755 | 0.924 | 0.058 | *a*b* | 0.148 | 0.301 | -0.214 | 0.989 |
|  |  |  |  |  | *b* | -0.060 | 0.089 | 0.502 |  |  |  |  |  |
| MHR | *a* | -0.069 | 0.060 | 0.250 | *c’* | 1.941 | 0.931 | 0.037 | *a*b* | -0.109 | 0.178 | -0.565 | 0.135 |
|  |  |  |  |  | *b* | 1.578 | 1.130 | 0.163 |  |  |  |  |  |
| SII | *a* | -253.303 | 70.233 | 0.001 | *c’* | 2.116 | 0.972 | 0.030 | *a*b* | -0.283 | 0.314 | -1.027 | 0.268 |
|  |  |  |  |  | *b* | 0.001 | 0.001 | 0.228 |  |  |  |  |  |
| SIRI | *a* | 0.074 | 0.238 | 0.755 | *c’* | 1.873 | 0.950 | 0.049 | *a*b* | 0.050 | 0.177 | -0.291 | 0.449 |
|  |  |  |  |  | *b* | 0.667 | 0.265 | 0.012 |  |  |  |  |  |

Boot LLCI/ULCI: the bootstrap method was used to test the mediating effect. The sample size was 5000, and the Upper Limit (UL) or Lower Limit (LL) Confidence Interval (CI) of the bootstrap B value for the mediating test was used. The non-standardized coefficient β and Standard Error (SE) are reported. If the confidence interval does not include zero, the relative indirect effect is statistically significant.

**Figure S1** **The path diagram of mediation analysis.** *c* means the total effect of independent variable *X* on dependent variable *Y*; *a* is the effect of *X* on mediating variable *M*; *b* is the direct effect of mediating variable *M* on dependent variable *Y*; *c’* is the direct effect of *X* on *Y* after adjusting for mediator *M*. The value *a*b* means the indirect effect.


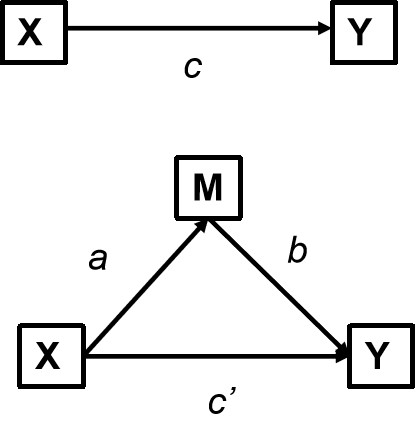

Supplement: Supplementary file 1 [file mmc1.docx]
